# A method for quantification of exportin-1 (XPO1) occupancy by Selective Inhibitor of Nuclear Export (SINE) compounds

**DOI:** 10.18632/oncotarget.6495

**Published:** 2015-12-07

**Authors:** Marsha L. Crochiere, Erkan Baloglu, Boris Klebanov, Scott Donovan, Diego del Alamo, Margaret Lee, Michael Kauffman, Sharon Shacham, Yosef Landesman

**Affiliations:** ^1^ All authors are current or former employees of Karyopharm Therapeutics Inc., Newton, MA, 02459 U.S.A

**Keywords:** selinexor, export, occupancy, cancer, resistance

## Abstract

Selective Inhibitor of Nuclear Export (SINE) compounds are a family of small-molecules that inhibit nuclear export through covalent binding to cysteine 528 (Cys528) in the cargo-binding pocket of Exportin 1 (XPO1/CRM1) and promote cancer cell death. Selinexor is the lead SINE compound currently in phase I and II clinical trials for advanced solid and hematological malignancies. In an effort to understand selinexor-XPO1 interaction and to establish whether cancer cell response is a function of drug-target engagement, we developed a quantitative XPO1 occupancy assay. Biotinylated leptomycin B (b-LMB) was utilized as a tool compound to measure SINE-free XPO1. Binding to XPO1 was quantitated from SINE compound treated adherent and suspension cells *in vitro*, dosed *ex vivo* human peripheral blood mononuclear cells (PBMCs), and PBMCs from mice dosed orally with drug *in vivo*. Evaluation of a panel of selinexor sensitive and resistant cell lines revealed that resistance was not attributed to XPO1 occupancy by selinexor. Administration of a single dose of selinexor bound XPO1 for minimally 72 hours both *in vitro* and *in vivo*. While XPO1 inhibition directly correlates with selinexor pharmacokinetics, the biological outcome of this inhibition depends on modulation of pathways downstream of XPO1, which ultimately determines cancer cell responsiveness.

## INTRODUCTION

A hallmark of all cancers regardless of their mutational status or histopathology is the dysregulation of the function of tumor suppressor proteins (TSPs) and growth regulatory proteins (GRPs) [1]. This malfunction is often directly attributed to the over expression or increased activity of exportin 1 (XPO1/CRM1) [2]. XPO1 serves as the nuclear export chaperone for over 200 different cargo proteins [3, 4]. The interaction between XPO1 and the activated small G-protein Ran (Ran-GTP) in the nucleus facilitates the binding to cargo proteins containing a short amino acid sequence of hydrophobic residues called a nuclear export signal (NES). This binding leads to subsequent transport of the multimeric complex through the nuclear pore, hydrolysis of Ran-GTP, release of the cargo in the cytoplasm, and return of XPO1 to the nucleus [5–8]. In cancer, over expression of XPO1 protein results in an aberrant distribution of TSPs to the cytoplasm where they are rendered ineffective at surveying genomic damage and preventing abnormal cell growth [9–21]. Experiments with the natural product inhibitor of XPO1, leptomycin B (LMB), demonstrated high potency and cytoxicity *in vitro* and *in vivo* [22], but this compound failed in the clinic due to poor tolerability [23]. In an effort to target this dysfunctional mechanism common to many cancer types, Karyopharm Therapeutics has developed Selective Inhibitor of Nuclear Export (SINE) compounds which bind to and inhibit XPO1 function [24]. SINE compounds are slowly reversible small molecule inhibitors that covalently bind to the cysteine 528 (Cys528) in the cargo binding pocket of XPO1, block the binding of NES cargo and hence prevent XPO1 from removing cargo from the nucleus [18, 25]. SINE compounds have been shown to effectively block nuclear export of many major TSPs and GRPs including p53, p21, FOXO, IκB, and survivin, both *in vitro* and *in vivo* [reviewed in [26]]. In cancer cells nuclear retention of TSPs by SINE compounds results in cell cycle arrest, inhibition of proliferation, initiation of apoptosis [reviewed in [27]], and prevention of DNA damage repair (Kashyap et al. 2014 EORTC poster, manuscript in preparation). Normal cells, however, are resistant to apoptotic effects of SINE compounds and typically undergo cell cycle arrest in the presence of these compounds [25]. These findings have made SINE compounds attractive therapies for a wide variety of solid and hematological malignancies [see [27] for review], as well as for the treatment of non-cancer indications with enhanced nuclear export activity [28–30].

Selinexor (KPT-330), the first clinical SINE compound, is currently being evaluated in Phase I and II clinical trials in many different cancer indications (see http://clinicaltrials.gov for details). To date, > 1000 patients have been dosed with selinexor and the drug has shown good tolerability with manageable side effects. Selinexor pharmacokinetic (PK) analysis in humans has shown that the drug has a T_max_ of ∼4 hours and near complete plasma clearance by ∼24 hours post-dose. In an effort to identify patients that would respond to selinexor treatment, we developed an assay that evaluates drug-target interaction from patient blood samples. In theory, this assay could be used to correlate a patient's response to selinexor to the level of XPO1 engagement. In the event that XPO1 from a particular patient was unable to bind selinexor then this individual could be offered an alternative therapy. Such an assay would be an ideal tool to screen patients to provide them with precision medicine and ensure they are receiving treatment that will be most effective for their malignancy.

## RESULTS

### Biotinylated LMB has anti-cancer properties that are similar to unmodified LMB

**Figure 1:**
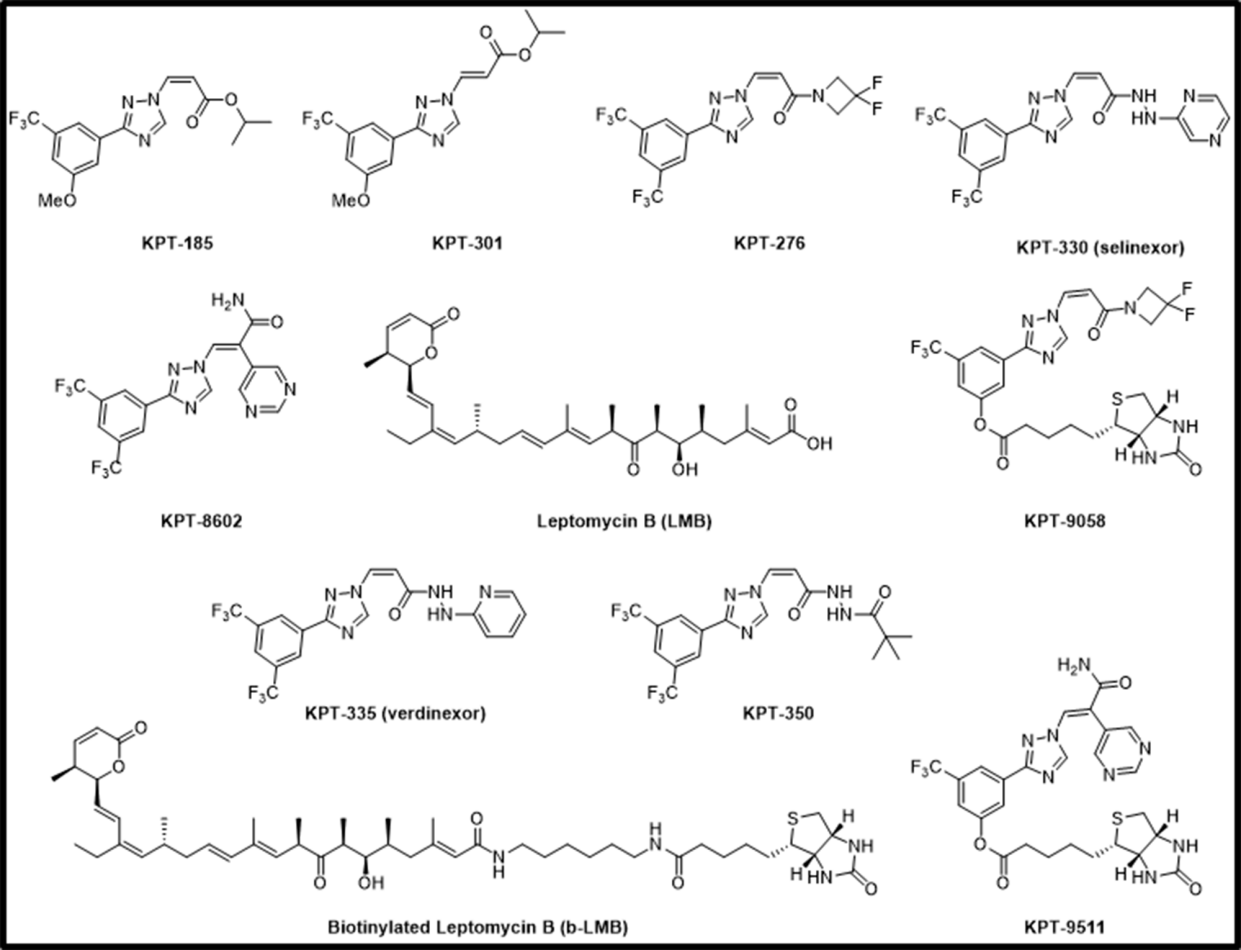
Chemical structures of SINE compounds KPT-185, KPT-301, KPT-276, KPT-330, KPT-8602, KPT-9058, KPT-335, KPT-350, KPT-9511, and leptomycin B (LMB) and biotinylated LMB (b-LMB)

To determine whether a patient's response to selinexor therapy could be predicted from evaluating drug-target interaction, we developed an assay to quantify XPO1 occupancy by SINE compounds. For this assay we utilized biotinylated LMB (b-LMB) [31] as a tool compound to quantify the amount of XPO1 bound by SINE compounds (Figure 1). b-LMB was synthesized by coupling a biotin tag to LMB. We compared the inhibition of nuclear export and the cytotoxicity of b-LMB to that of the unmodified LMB. We first evaluated the nuclear retention of the XPO1 cargo protein Rev-GFP (HIV-Rev fused to the cAMP-dependent Protein Kinase Inhibitor (PKI) nuclear export signal) stably expressed in U2OS cells treated with selinexor, LMB or b-LMB (Table 1). We then quantified the effects of these compounds on cell death in several cancer cell lines using an MTT cytotoxicity assay (Table 2). The ability of b-LMB to induce nuclear retention of Rev-GFP in U2OS cells was similar to unmodified LMB (IC_50_ 0.11 nM vs. 0.16 nM, respectively; Table 1) and was 360-fold more potent than selinexor (IC_50_ 40 nM; Table 1). Although b-LMB was less cytotoxic than unmodified LMB (∼6 – 30-fold), it proved to be more potent than selinexor in all cell lines tested (Table 2). These results indicated that b-LMB has anti-cancer activity that is on par with unmodified LMB and is more potent than selinexor, allowing for further development as a tool compound for the XPO1 occupancy assay.

**Table 1:** b-LMB inhibits nuclear export of the XPO1 cargo protein Rev-GFP in stably transfected U2OS cells similarly to unmodified LMB

Compound	Rev-GFP (nM)
Selinexor	40
LMB	0.16
b-LMB	0.11

**Table 2:** b-LMB has anti-cancer activity that is less potent than unmodified LMB in a cellular cytotoxicity assay

Cell Line	Selinexor IC_50_ (nM)	LMB IC_50_ (nM)	b-LMB IC_50_ (nM)
MM.1S	20	0.46	3.1
HCT-116	50	0.19	6.0
AML2	90	0.39	2.53
AML3	270	0.68	4.68

### XPO1 can bind b-LMB in a dose-dependent manner

**Figure 2:**
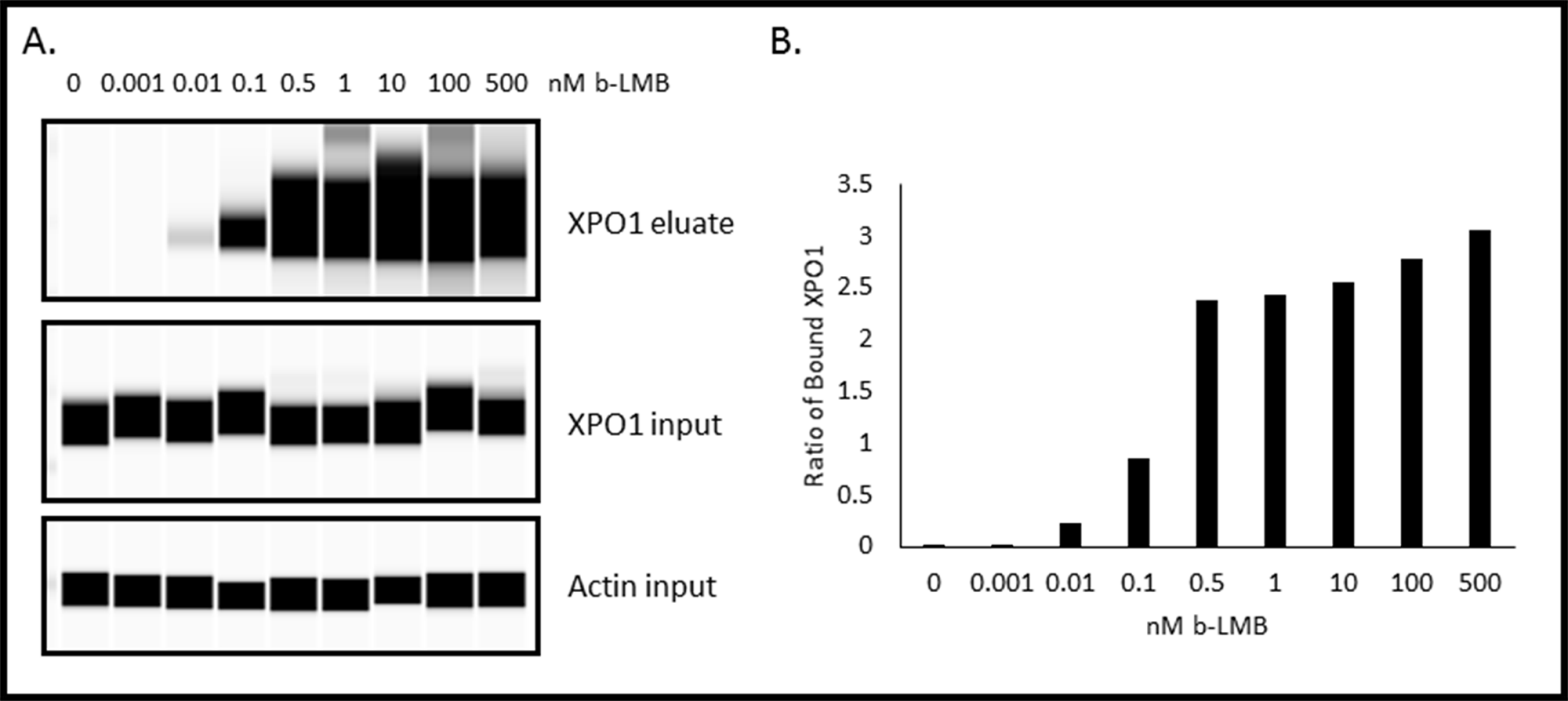
b-LMB binds XPO1 in a dose-dependent manner in MM.1S cells MM.1S cells were treated with increasing concentrations of b-LMB and evaluated in the XPO1 occupancy assay. **A.** Representative digital western blot images for eluates and inputs probed with antibodies to XPO1 and β-actin. **B.** Plot of the ratio of bound XPO1 as a function of b-LMB concentration. The ratio of bound XPO1 was calculated by dividing the mean peak area of b-LMB bound XPO1 detected in the eluate by the mean peak area of all XPO1 detected in the input lysate.

b-LMB was tested for the ability to bind XPO1 protein. First, a dose-response curve was generated by exposing MM.1S cells to increasing concentrations of b-LMB (0 – 500 nM) for 1.5 hours in culture. The cells were then collected, lysed, and a portion of the lysate (input) was reserved prior to incubating the remaining lysate with streptavidin-tagged beads overnight. After extensive washes, b-LMB bound XPO1 was eluted from the beads, and XPO1 from both input and bead eluate was detected and quantified by Simple Western on Peggy Sue. Simple Western allows for the chemiluminescent detection of proteins from small volumes of samples that are run through a matrix-filled capillary. The signal is detected by a CCD camera which generates a digital image and then reports quantitative results. Figure 2A is a representative digital western for XPO1 detected in the eluate and input samples, with β-actin as a loading control. XPO1 was detected in the eluate from cells treated with as little as 0.01 nM of b-LMB, while equal levels of XPO1 and actin proteins were detected in all input samples tested. The protein signals for XPO1 detected by Simple Western in Figure 2A were reported as mean peak areas and were used to compare levels of XPO1 present in each sample to produce a graphical representation of the ratio of free XPO1 detected at each concentration of b-LMB (Figure 2B). “Ratio of bound XPO1” is defined as the ratio of the mean peak area of b-LMB bound XPO1 detected in the eluate compared to the mean peak area of all XPO1 detected in the input lysate, while “free-fraction” refers to the amount of XPO1 not occupied by SINE compound, as described later. Increasing levels of bound XPO1 were detected with increasing concentrations of b-LMB (Figure 2B). The amount of b-LMB necessary to occupy 50% and 90% of XPO1 was calculated from the ratio of free XPO1 versus b-LMB concentration in MM.1S cells, which was 0.27 +/− 0.11 and 2.53 +/− 1.72 nM (*n* = 3), respectively.

### Occupation of XPO1 by SINE compounds can be quantitated

**Figure 3:**
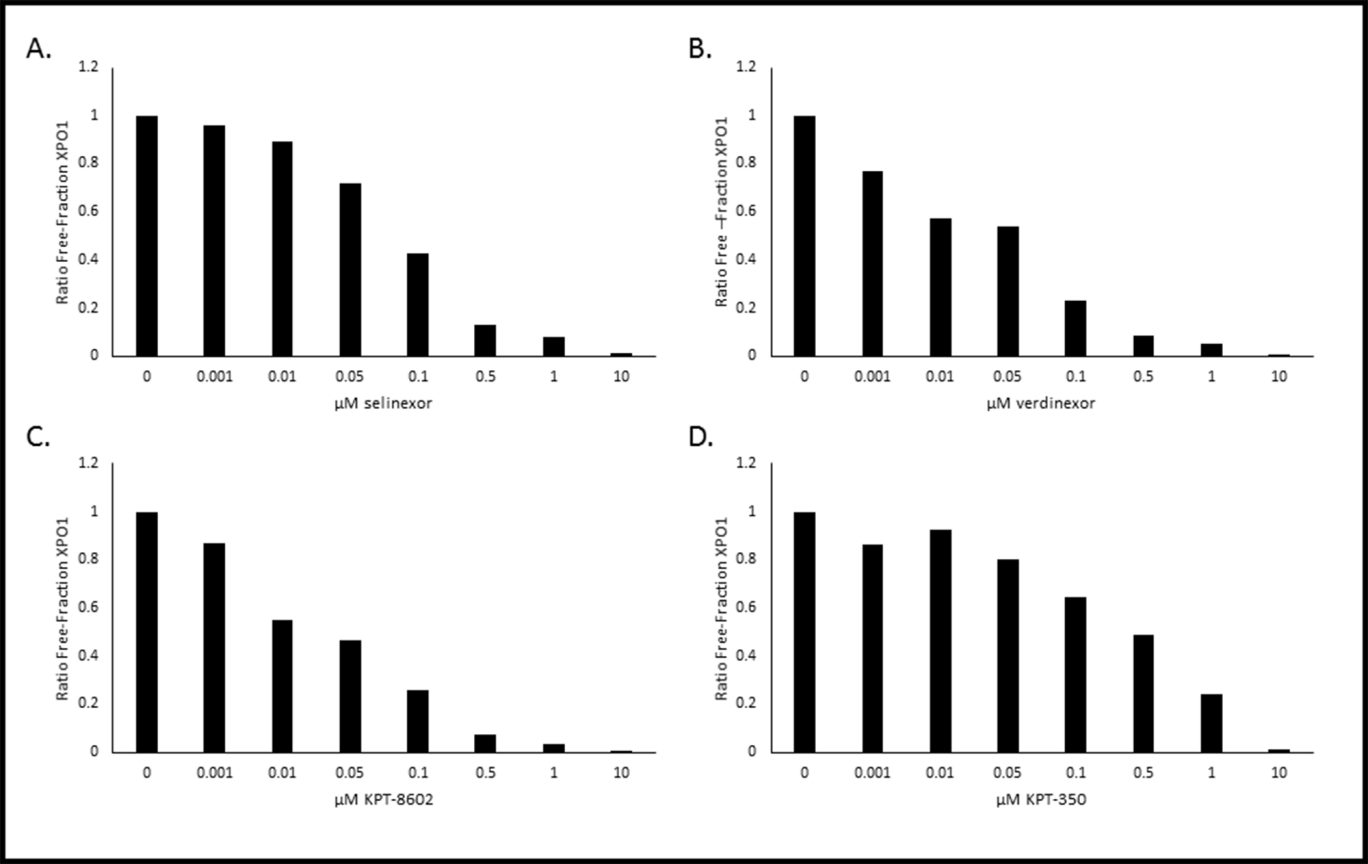
SINE compounds occupy XPO1 in a dose-dependent manner in MM.1S cells with KPT-350 being the least potent MM.1S cells were treated with increasing concentrations of either selinexor **A.** verdinexor **B.** KPT-8602 **C.** or KPT-350 **D.** and were subsequently processed for the XPO1 occupancy assay. Representative plots for the ratio of free-fraction XPO1 for each SINE compound is shown which was calculated by dividing the mean peak areas of XPO1 occupied by b-LMB (from the eluate) by the total amount of XPO1 (from the input), normalized to the corresponding SINE compound-free control.

The next step was to evaluate whether selinexor or other SINE compounds could dose-dependently bind to XPO1. MM.1S cells were treated with increasing concentrations (0 – 10 μM) of SINE compounds selinexor, verdinexor (KPT-335), KPT-8602, or KPT-350 for one hour prior to treatment with 1 nM b-LMB, the amount found to reach a plateau in MM.1S cells (Figure 2B) as well as the amount tested by Sakakibara et al [32], for 1.5 hours. The cells were then collected and processed for the XPO1 occupancy assay. Figure 3 shows a graphical representation of an example of the ratios of free-fraction XPO1 (i.e. unbound by SINE) versus SINE compound concentration. For these assays, the “ratio of free-fraction XPO1” is calculated by dividing the mean peak areas of XPO1 occupied by b-LMB (from the eluate) by the total amount of XPO1 (from the input). The ratio of free-fraction XPO1 is indicative of the amount of SINE compound bound to Cys528 of XPO1; a lower ratio of free-fraction XPO1 means more XPO1 is occupied by SINE compound and vice versa. All four SINE compounds bound in a dose-dependent manner to XPO1 in the XPO1 occupancy assay. The average 50% and 90% occupancy values were calculated for each SINE compound and were compared to their Rev-GFP values (Table 3). Verdinexor, KPT-8602, and selinexor were similarly effective at occupying XPO1 while KPT-350 was less potent in the XPO1 occupancy assay. This is consistent with the higher Rev-GFP IC_50_ value for KPT-350, indicating that higher KPT-350 concentration is needed to achieve entrapment of REV-GFP relative to the other SINE compounds (Table 3). As a negative control, the XPO1 occupancy assay was also performed with KPT-301, the inactive, trans isomer of KPT-185 [[18, 25]; Figure 1], that does not have anti-cancer activity [15, 33, 34]. Treating MM.1S cells with increasing concentrations (0 – 10 μM) of KPT-301 followed by 1 nM b-LMB showed that no XPO1 bound to KPT-301, demonstrating the specificity of the active SINE compounds for XPO1 occupancy (data not shown). Together, these results indicate that b-LMB can be effectively utilized as an *in vitro* tool compound to evaluate the occupancy of all XPO1 inhibitors.

**Table 3:** Rev-GFP values correlate to XPO1 occupancy values in MM.1S cells treated with different SINE compounds

SINE Compound	Rev-GFP IC_50_ (μM)	Avg 50% Occupancy (μM)	Avg 90% Occupancy (μM)
Verdinexor	0.01	0.04 +/− 0.03 (*n* = 3)	0.34 +/− 0.08 (*n* = 3)
KPT-8602	0.04	0.06 +/− 0.06 (*n* = 2)	1.1 +/− 1.03 (*n* = 2)
Selinexor	0.04	0.08 +/− 0.01 (*n* = 2)	0.52 +/− 0.05 (*n* = 2)
KPT-350	0.17	0.63 +/− 0.29 (*n* = 3)	6.45 +/− 3.36 (*n* = 3)

### PBMCs can be utilized as a sample source to measure XPO1 occupancy by selinexor

**Figure 4:**
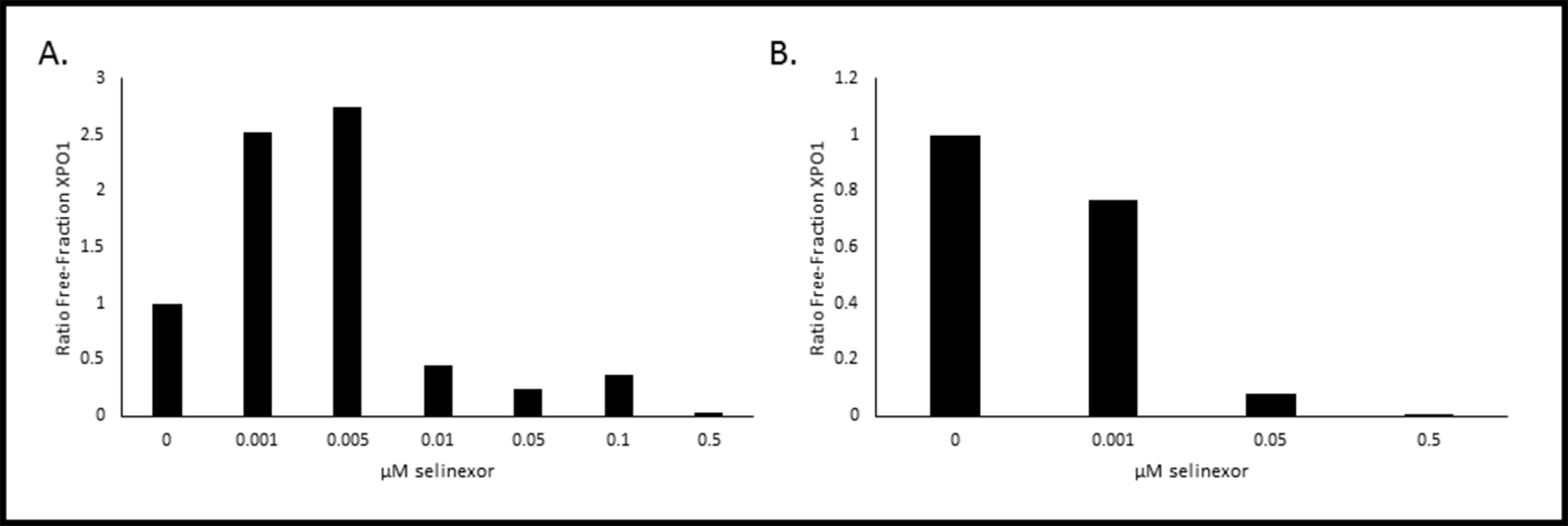
Selinexor binds to XPO1 in both freshly isolated or revived from cryogenic preservation human PBMCs *ex vivo* PBMCs were isolated and immediately treated with increasing concentrations of selinexor and processed for the XPO1 occupancy assay **A.** or were viably frozen, thawed, then treated with increasing concentrations of selinexor and processed for the XPO1 occupancy assay **B.** Representative plots for the ratio of free-fraction XPO1 for each condition is shown which was calculated by dividing the mean peak areas of XPO1 occupied by b-LMB (from the eluate) by the total amount of XPO1 (from the input), normalized to the corresponding selinexor-free control.

Our goal was to use this method to quantify XPO1 occupancy in patient tumor biopsies. However, after numerous attempts we found that this assay could only measure XPO1 occupancy in living cells with intact membranes. Therefore, in addition to using this method on cultured monolayer cells that can be trypsinized into suspension cells, we also used the method on hematological suspension cells. Development of this method in hematological cells derived from selinexor patient peripheral blood or bone marrow biopsies required additional optimization. We envisioned collection of biopsies remotely in hospitals then shipment to a central laboratory performing the assay. For this we optimized the assay for measuring XPO1 occupancy in human PBMCs. Therefore it would require conditions for preserving these cells cryogenically at the clinic collecting the samples. In order to evaluate this process, the XPO1 occupancy assay was performed *ex vivo* on PBMCs isolated from pooled healthy donor blood, where half of the PMBCs were assayed fresh while the other half were cryopreserved (Figure 4). First, the assay was performed on the freshly isolated PBMCs (Figure 4A). Similar to the method described above, PBMCs were incubated with increasing concentrations of selinexor (0 – 0.5 μM) for 1 hour prior to a 1 hour treatment with 1 nM b-LMB. In freshly isolated PBMCs, 50% XPO1 occupancy occurred at 0.01 μM selinexor, while 90% occupancy occurred at > 0.5 μM (Table 4), similar to results obtained in MM.1S cells. Since patient samples must be isolated and viably frozen at the clinic prior to being tested, the assay was performed on PBMCs that were revived after being viably frozen. For this, the other half of the pooled healthy donor PBMCs were cryopreserved, revived and assayed (Figure 4B). To allow sufficient time for recovery from cryopreservation, revived PBMCs were treated with increasing concentrations of selinexor (0 – 0.5 μM) for 4 hours, subsequently washed to remove the drug, then treated with 1 nM b-LMB for 1 hour prior to the XPO1 occupancy assay. In revived PBMCs, 50% XPO1 occupancy occurred at the same concentration of selinexor as that of freshly isolated PBMCs (Table 4). These *ex vivo* assays demonstrated that PBMCs isolated from patients in the clinic and viably frozen would be a suitable source of material to perform the XPO1 occupancy assay.

**Table 4:** XPO1 occupancy values are similar regardless of whether PBMCs are processed immediately after isolation or revived from cryogenic preservation

Human PBMCs	50% Occupancy (μM)	90% Occupancy (μM)
Fresh	0.01	> 0.5
Revived	0.01	0.04

**Figure 5:**
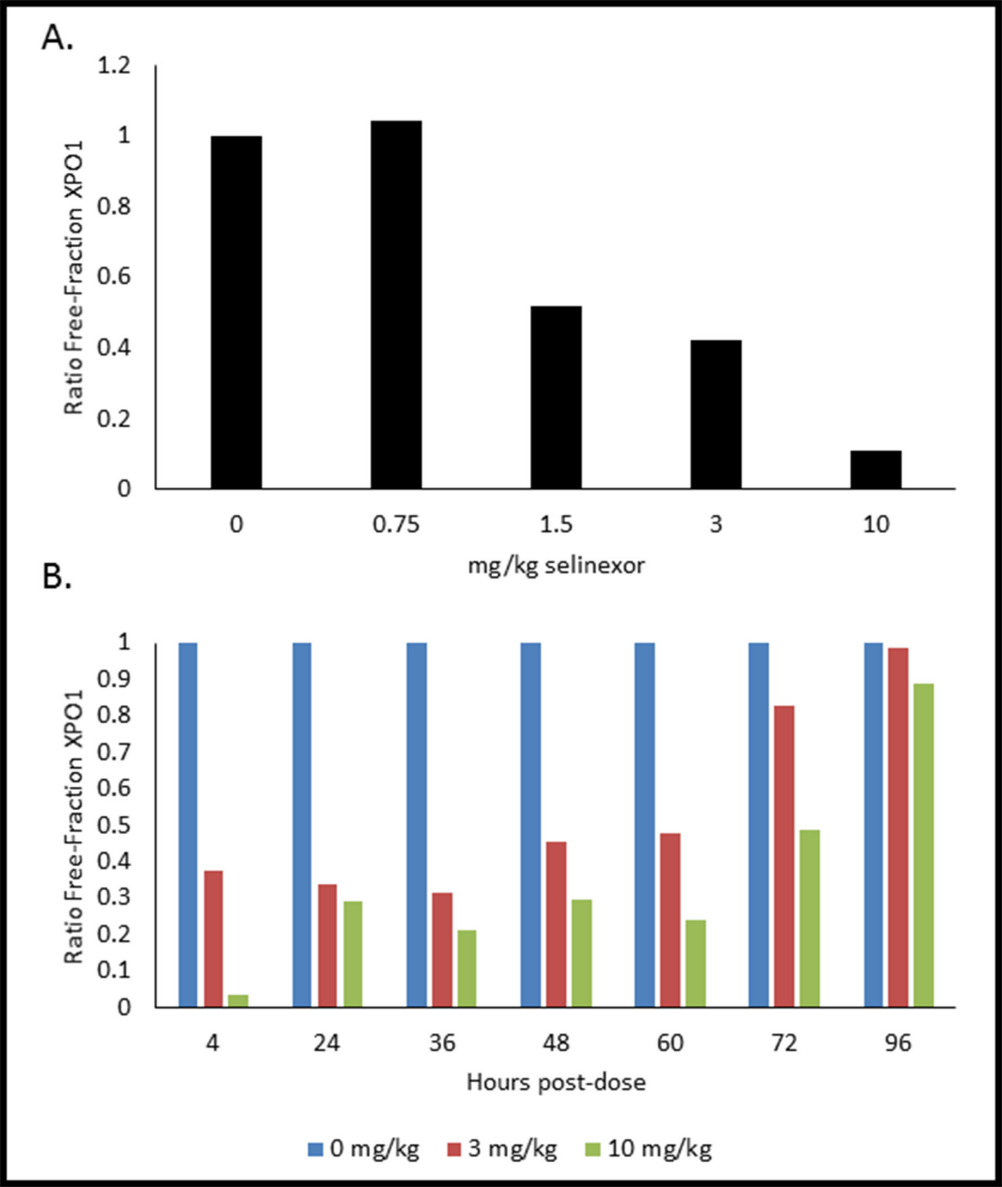
PMBCs from mice dosed with selinexor show dose- and time-dependent responses of XPO1 occupancy **A.** Mice (*n* = 4 per dose) were administered a single oral gavage of either 0, 0.75, 1.5, 3, or 10 mg/kg of selinexor for 4 hours, were terminally bled, and then PBMCs were processed for the XPO1 occupancy assay. Ratio of free-fraction XPO1 versus selinexor dose is shown (see how calculated below). **B.** Mice (*n* = 4 per dose per time point) were dosed once with either 0, 3, or 10 mg/kg selinexor and terminally bled at 4, 24, 36, 48, 60, 72, and 96 hours post-dose. PBMCs were processed for the XPO1 occupancy assay. Ratio of free-fraction XPO1 versus selinexor dose for each time point is shown which was calculated by dividing the mean peak areas of XPO1 occupied by b-LMB (from the eluate) by the total amount of XPO1 (from the input), normalized to the corresponding selinexor-free control.

Based on these *ex vivo* results, we sought to determine whether the XPO1 occupancy assay could be performed on PBMCs exposed to selinexor *in vivo* (Figure 5). Mice were orally administered vehicle, 0.75, 1.5, 3, or 10 mg/kg of selinexor and 4 hours later the mice were terminally bled. In order to have enough PBMCs to assay, the blood from the mice for each dose (*n* = 4) was pooled and PBMCs were immediately isolated. Mouse PBMCs were incubated with 1 nM b-LMB for 1.5 hours *ex vivo* then collected and prepared for the XPO1 occupancy assay. Increasing doses of selinexor corresponded to increasing occupancy of XPO1, as indicated by a decrease in the ratio of free-fraction XPO1 (Figure 5A). The amount of selinexor required to occupy 50% of XPO1 at 4 hours post-dosing was 1.84 mg/kg, while the amount to achieve 90% occupancy was > 10 mg/kg.

### Single dose administration of selinexor results in sustained XPO1 occupancy

Using similar conditions, we sought to determine the duration of XPO1 occupancy by SINE compounds in mice. Once again mice were orally administered a single dose of either placebo, 3, or 10 mg/kg of selinexor. The mice were then terminally bled at 4, 24, 36, 48, 60, 72, and 96 hours post dose (*n* = 4 mice per dose per time point). Blood for each dose at each time point was pooled and PMBCs were isolated. Mouse PBMCs were incubated with 1 nM b-LMB for 1.5 hours *ex vivo* before being processed for the XPO1 occupancy assay. Both 3 and 10 mg/kg had time-dependent increases in the ratio of free-fraction XPO1, with 50% XPO1 occupancy being sustained for up to 72 hours post-treatment (Figure 5B). Interestingly, full saturation of XPO1 was already achieved between 4–24 hours at the lower dose of 3 mg/kg. These *in vivo* data demonstrate that it is possible to correlate the level of occupied XPO1 to the amount of drug administered and that the binding of selinexor to XPO1 is robust and sustained for up to 72 hours after a single oral dose.

### Functional XPO1 inhibition correlates to XPO1 occupancy by selinexor

**Figure 6:**
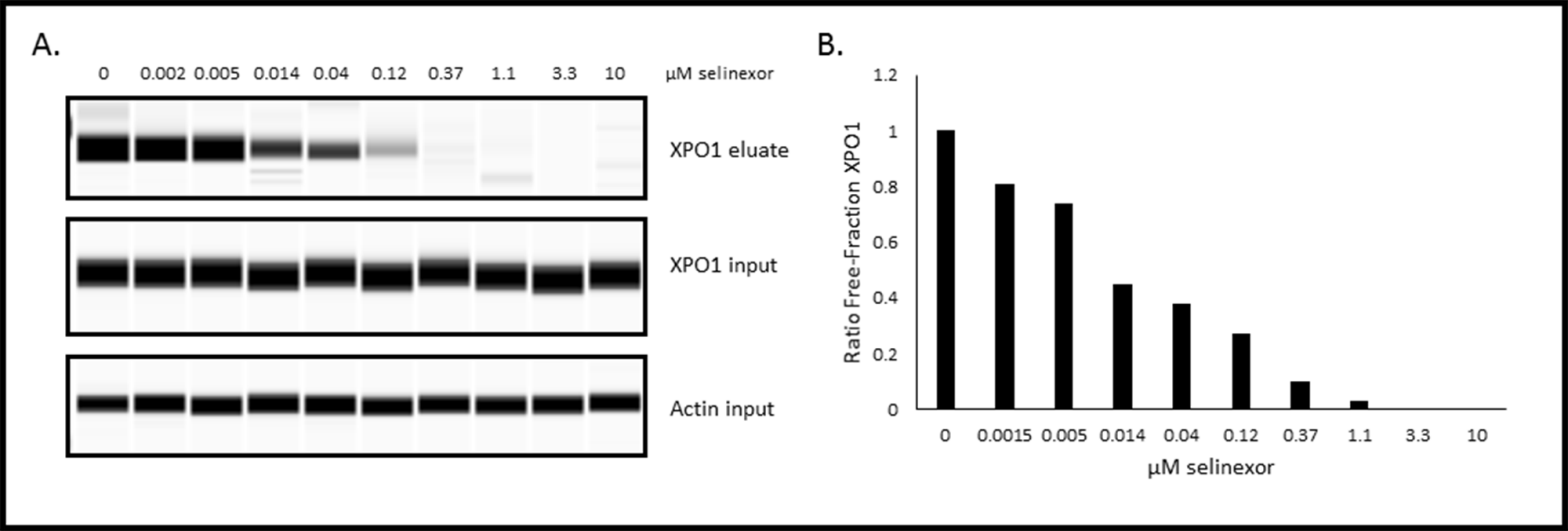
Selinexor binds to XPO1 in a dose-dependent manner in Rev-GFP U2OS cells in the XPO1 occupancy assay **A.** Digital western blot images for eluates and inputs from Rev-GFP U2OS cells that were treated with serially increasing concentrations of selinexor and processed for the XPO1 occupancy assay. **B.** Plot of the ratio of free-fraction XPO1 for each selinexor concentration which was calculated by dividing the mean peak areas of XPO1 occupied by b-LMB (from the eluate) by the total amount of XPO1 (from the input), normalized to the corresponding selinexor-free control.

Although the *in vivo* data indicated that selinexor remained bound to XPO1 for up to 72 hours, it did not address the question of whether XPO1 was continuously inhibited over this period of time. To test whether time-dependent XPO1 occupancy by selinexor correlated with inhibition of activity, we used the U2OS Rev-GFP cells in the XPO1 occupancy assay. First, these cells were treated with increasing concentrations of selinexor (0–10 μM) for 1 hour, followed by 1 nM b-LMB treatment for 1.5 hours, and then analyzed by the XPO1 occupancy assay (Figure 6). Figure 6A shows the XPO1 protein levels detected from eluates and inputs from the U2OS cells in each treatment group (β-actin is a loading control). Figure 6B is the graphical representation of the ratio of free-fraction XPO1 for each concentration of selinexor from Figure 6A. Selinexor bound dose-dependently to XPO1 in a similar fashion to MM.1S cells, with 50% of XPO1 occupancy occurring at 0.02 μM and 90% at 0.48 μM (compare to values in Table 3).

**Figure 7:**
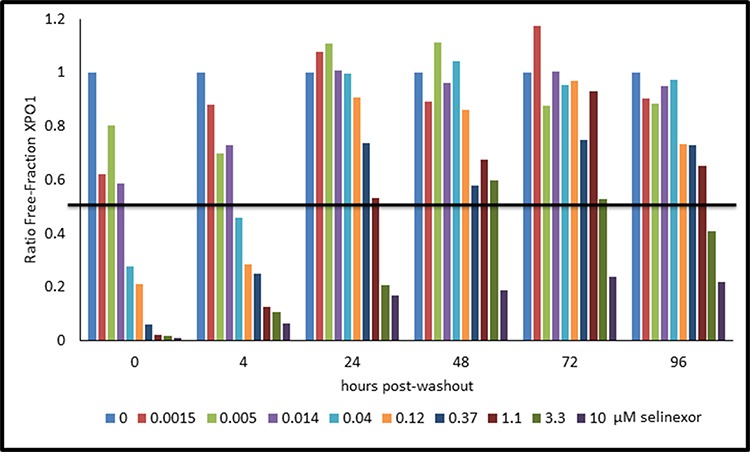
Selinexor – XPO1 interaction persists with time in a dose-dependent manner post drug wash-out in Rev-GFP U2OS cells The ratio of free-fraction XPO1 was calculated from cells that were dosed for 4 hours with increasing amounts of selinexor followed by drug wash out and collection at 4, 24, 48, 72, and 96 hours post wash-out. Horizontal line demarks the position where half of XPO1 is occupied by selinexor. The ratio of free-fraction XPO1 was calculated by dividing the mean peak areas of XPO1 occupied by b-LMB (from the eluate) by the total amount of XPO1 (from the input), normalized to the corresponding selinexor-free control for each time point.

Since Rev-GFP cells were adequate for the XPO1 occupancy assay, these cells could be used to test the correlation between drug-target interaction and inhibition of protein activity. Rev-GFP cells were treated with 3 fold serial dilutions of selinexor (starting from 10 μM) for 4 hours, the time at which maximum Rev-GFP inhibition occurs. Cells were washed to remove the compound, replenished with fresh compound-free media, and then assayed at 0, 4, 24, 48, 72, and 96 hours post wash-out. At each time point the cells were prepared in duplicate such that one set was treated with 1 nM b-LMB and used for the XPO1 occupancy assay (Figure 7, Table 5), while the other set was prepared for immuno-fluorescent detection of Rev-GFP (Table 5). Figure 7 is the graphical representation for the ratio of free-fraction XPO1 plotted for each concentration of selinexor at each time point post wash-out. The horizontal line demarcates the 50% XPO1 occupancy by selinexor. Selinexor occupied XPO1 in a dose-dependent manner at 0, 4, and 24 hours post wash-out (Figure 7). From 48 to 96 hours post wash-out 3 and 10 μM selinexor resulted in persistent XPO1 occupation (Figure 7). 50% occupancy of XPO1 was maximized at 3 μM of selinexor at 48 hours and longer (Table 5). Similar to the PBMCs time-course from treated mice (Figure 5B), a single dose equivalent of selinexor demonstrates robust and sustained target interaction with full saturation of XPO1 occurring between 4–24 hours post-treatment both *in vivo* and *in vitro*. Wash-out of selinexor in the Rev-GFP assay showed that there was a decrease in inhibition of Rev nuclear export with time. The time course revealed a dose-dependent response to selinexor, with Rev-GFP being retained in the nucleus at IC_50_ values of 1.3 μM selinexor at 48 hours, 6.8 μM at 72 hours, and 11.4 μM at 96 hours (Table 5). Comparison of these Rev-GFP IC_50_ values to the corresponding 50% XPO1 occupancy values suggests that administration of a low dose of selinexor more frequently is as effective as administration of a higher dose of selinexor given less often. These results indicate that there is a correlation between the amounts of drug administered to the persistence of target binding coupled with inhibition of protein activity.

**Table 5:** Rev-GFP IC_50_ and XPO1 occupancy values for Rev-GFP U2OS cells assayed in the dose-response, time-course, wash-out experiment

Hours post washout	RevGFP Assay IC_50_ (μM)	50% Occupancy (μM)	90% Occupancy (μM)
0	0.01	0.02	0.16
4	0.02	0.04	2.87
24	0.5	1.06	> 10
48	1.3	2.95	> 10
72	6.8	3.49	> 10
96	11.4	2.17	> 10

### XPO1 occupancy by selinexor occurs to the same extent regardless of drug sensitivity

To test whether the XPO1 occupancy assay can predict sensitivity of cancer cells to selinexor treatment, it was performed on a panel of cell lines from many different types of both solid and hematological cancers each with various levels of selinexor sensitivity (by MTT, IC_50_ values). Table 6 lists each cell line tested in the XPO1 occupancy assay compared to the corresponding selinexor IC_50_ (sensitive to resistant). Regardless of sensitivity to selinexor by IC_50_ values, all cell lines tested had similar 50% and 90% occupancy values. However, when the XPO1 input areas for each cell type were averaged and then divided by the number of cells used for the assay, there was a strong correlation between the relative amount of XPO1 protein per cell and the sensitivity of that cell type to selinexor by the MTT assay. This result indicates that while loading of the drug into the XPO1 target occurs by the same kinetics in sensitive and resistant cells, high XPO1 protein levels itself could predict drug resistance.

**Table 6:** Cell lines with varying sensitivities to selinexor (IC_50_ values) have similar XPO1 occupancy values

Cell Line	Selinexor IC_50_ (μM)	50% Occupancy selinexor (μM)	90% Occupancy selinexor (μM)	Relative XPO1 protein level per cell
MM.1S	0.02	0.07	0.48	0.5
MV-4–11	0.02	0.04	0.61	0.06
HCT-116	0.05	0.15	0.76	0.52
HT1080	0.07	0.44	1.35	1.74
AML2	0.09	0.01	0.22	0.07
AML3	0.27	0.04	0.46	0.01
HEL	0.35	0.12	0.31	0.1
Kasumi-6	0.53	0.14	0.57	0.13
THP-1	1.06	0.1	0.94	0.44
HT1080 resistant	2.4	0.4	1.67	1.99
A549	3	0.09	0.56	0.3
UCH1	7	0.15	0.65	2.18
UCH2	30	0.27	0.89	2.84
LS174T	>10	0.11	0.38	0.32
ASPS-KY	>10	0.24	0.72	2.65

### Biotinylated SINE compounds are tools for evaluating SINE-XPO1 occupancy

**Figure 8:**
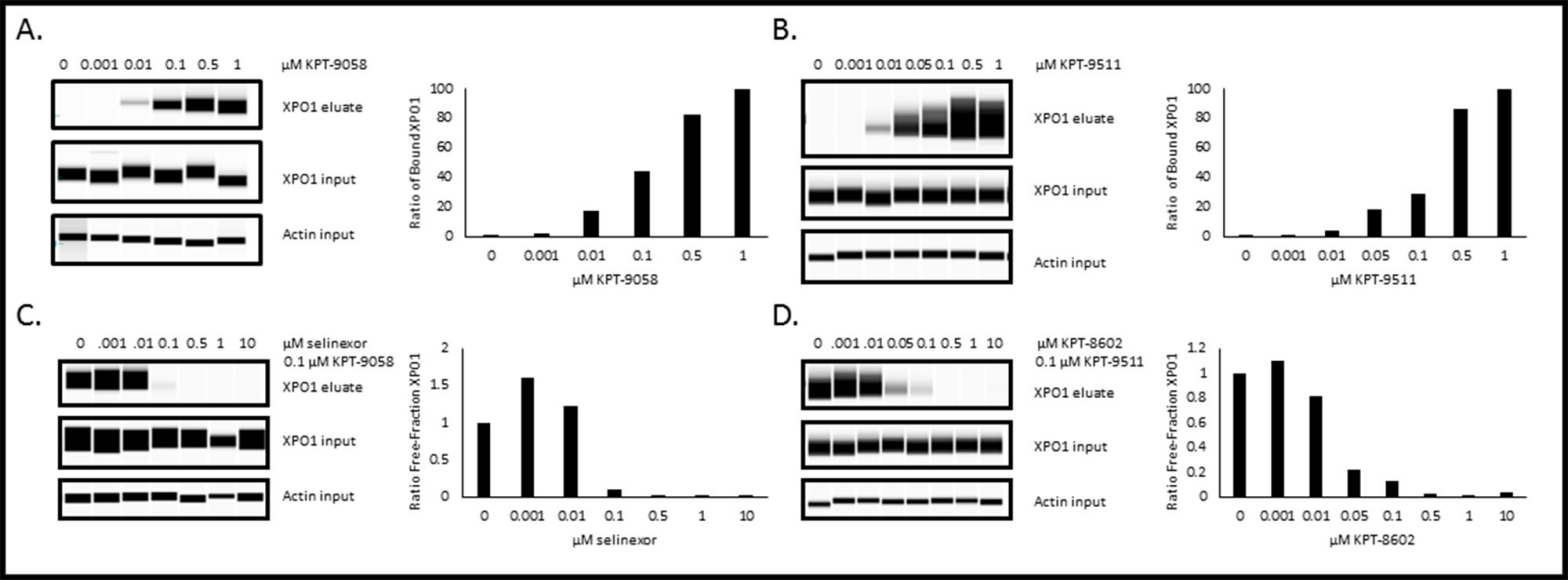
Biotinylated SINE compounds bind XPO1 in a dose-dependent manner and unmodified SINE compounds bind XPO1 at a 1:1 ratio to biotinylated SINE compounds in MM.1S cells MM.1S cells were treated with increasing concentrations of either KPT-9058 **A.** or KPT-9511 **B.** and processed for the XPO1 occupancy assay. MM.1S cells were treated with increasing concentrations of either selinexor **C.** or KPT-8602 **D.** followed by treatment with 0.1 μM biotinylated SINE compound and processed for the XPO1 occupancy assay. Digital western blot images for eluates and inputs as well as plots for the ratio of free-fraction XPO1 versus SINE compound concentration are shown. The ratio of free-fraction XPO1 was calculated by dividing the mean peak areas of XPO1 occupied by b-LMB (from the eluate) by the total amount of XPO1 (from the input), normalized to the corresponding SINE compound-free control.

Despite the fact that the XPO1 occupancy assay may not be able to predict patient response to selinexor, the assay could potentially be used to measure drug delivery and XPO1 inhibition. Since b-LMB is not pharmacologically relevant to SINE compounds, we next used this assay to compare the ability of other biotin-tagged SINE compounds to bind to XPO1. Initially biotin was added to selinexor, but the modification significantly impaired the potency of the compound and therefore was not able to be utilized in the assay. Next, additional biotinylated SINE compounds were developed as a more relevant comparison to untagged SINE compound for patient samples. KPT-9085, the biotinylated version of KPT-276, and KPT-9511, the biotinylated version of KPT-8602 (Figure 1) were less potent in Rev-GFP and in MTT assays compared to their unmodified counterparts (Table 7). KPT-9058 was ∼18-fold less potent in Rev-GFP compared to KPT-276 (961 vs. 53 nM) and ∼6-fold less cytotoxic (110 nM vs. 18 nM). KPT-9511 was ∼5 fold less potent in Rev-GFP compared to KPT-8602 (230 nM vs 42 nM) and ∼15 fold less cytotoxic (280 vs 19 nM). Both biotinylated compounds, however, showed dose-dependent binding to XPO1 and a binding ratio of 1:1 with their unmodified counterparts in the XPO1 occupancy assay (Figure 8, Table 8). MM.1S cells were treated with increasing concentrations of KPT-9058 (Figure 8A) or KPT-9511 (Figure 8B) and the corresponding 50% occupancy values were determined to be 0.12 μM for KPT-9058 and 0.18 μM for KPT-9511. MM.1S cells were then treated with increasing concentrations of either selinexor (Figure 8C) or KPT-8602 (Figure 8D) followed by treatment with 0.1 μM of either KPT-9058 or KPT-9511. 50% and 90% XPO1 occupancy values were similar for each unmodified SINE compound/biotinylated SINE compound pair (Table 8). Selinexor had 50% and 90% XPO1 occupancy values of 0.03 and 0.1 μM with KPT-9058, and KPT-8602 had 50% and 90% occupancy values of 0.02 and 0.11 μM with KPT-9511. For both selinexor and KPT-8602, the 90% occupancy values were equal to the amount of biotinylated SINE compound used for the assay, indicating a 1:1 ratio of binding of unmodified SINE compound compared to the biotinylated SINE compound. These data suggest that biotinylated SINE compounds can be used as tools to effectively evaluate the binding capacity of SINE compounds to XPO1 protein for research purposes.

**Table 7:** Rev-GFP and MTT IC_50_ values for unmodified and biotinylated SINE compounds

Compound	Rev GFP IC_50_ (nM)	MTT IC_50_ (nM)
KPT-276	53	18
KPT-9058 (biotinylated KPT-276)	961	110
KPT-8602	42	19
KPT-9511 (biotinylated KPT-8602)	230	280

**Table 8:** XPO1 occupancy values for MM.1S cells treated with biotinylated SINE compounds and unmodified SINE compounds with biotinylated SINE compounds

SINE Compound(s)	50% Occupancy (μM)	90% Occupancy (μM)
KPT-9058 (biotinylated KPT-276)	0.12	0.69
Selinexor/KPT-9058	0.03	0.10
KPT-9511 (biotinylated KPT-8602)	0.18	0.61
KPT-8602/KPT-9511	0.02	0.11

## DISCUSSION

Selinexor is currently being evaluated in Phase I and II clinical trials to treat patients with both solid and hematological malignancies. In addition to pharmacological measurements, it is clinically beneficial to develop an assay that will assess the level of target occupancy. Similar occupancy assays have been developed for other drug-target interactions such as ibrutinib/Bruton tyrosine kinase [35, 36] and MDX-1106/programmed death-1 [37] proteins. While pharmacokinetic studies indicate an availability of selinexor in peripheral blood and in certain tissues or tumor, the occupancy assay correlates direct binding of the drug to its target with dosing levels. Such correlative studies of target engagement have the power to help optimize clinical trial dose while not exceeding levels that may potentially induce harsh adverse events.

When we first developed the XPO1 occupancy assay, we hypothesized that this assay would explain the difference in cancer cell sensitivity to selinexor observed in cytotoxicity assays. We predicted that sensitive cells would have increased XPO1 occupancy at a lower drug concentration, and therefore utilize a patient's cancer cells to predict a positive response to selinexor. We found, however, that selinexor was capable of binding to XPO1 similarly in all cell types tested regardless of their sensitivity to selinexor. Hence this assay demonstrated that drug sensitivity was determined by factors downstream of drug-target interaction and possibly by overexpression of the XPO1 target. We also found that different SINE compounds bound XPO1 with a potency that correlated with their capacity to inhibit nuclear export by the Rev-GFP assay and to induce cell death by MTT assay. Additionally, we found that the binding of selinexor to XPO1 was prolonged and sustained whereby saturation was achieved at 4–24 hours and persisted for up to 72 hours both *in vitro* and *in vivo* after a single administration indicating that dosing of patients could be further optimized.

Although this assay successfully correlated occupancy of XPO1 to dosing level, this assay revealed no correlation of XPO1 occupancy to efficacy across a diverse panel of cell lines. Therefore, we can conclude that the difference in response to selinexor is downstream of XPO1 inhibition. We came to this same conclusion when we compared the parental HT1080 (fibrosarcoma) cell line to the *in vitro*-generated selinexor resistant counterpart [38]. Comparison of sensitive parental cells to resistant cells by microarray analysis revealed differences in pathways related to adhesion, apoptosis, and inflammation. Given the vast number of cargoes which rely on XPO1 for nuclear transport it is reasonable to suspect that selinexor response may be governed by the cellular context of signaling mechanisms downstream of nuclear export inhibition and the differential dependence of tumor cell survival on these downstream mechanisms in one type of cancer versus another. Indeed the concept of oncogene addiction is now known to play a role in growth and survival of certain tumor types, and exploitation of this concept has led to some successful precision cancer therapeutics [39, 40]. The same general principle may apply to XPO1 inhibition, where in a particular tumor cell context, cell survival is exquisitely dependent on the cytoplasmic localization (or reduced nuclear localization) of an XPO1 cargo. In particular this concept of differential dependence on XPO1 cargo localization based on cellular context is further supported by the observation that normal, non-malignant cells are more resistant to apoptosis induced by XPO1 inhibition than their malignant counterparts [25].

Our studies indicate that cancer cell response to selinexor therapy is determined by pathways downstream of XPO1 inhibition, and in at least 4 out of 7 selinexor resistant cell lines, could also be determined by overexpression of the XPO1 target. Such an overexpression enhances exclusion of essential TSPs and GRPs from the nucleus and supports unregulated cell division. This observation should be further tested in additional cell lines as well in biopsies of selinexor treated patients where drug response is documented.

The XPO1 occupancy assay could be used to determine direct inhibition of XPO1 in a specific tumor biopsies but only from intact and viable cells. To use this assay in biopsies of solid tumors additional optimization steps are needed. Optimization of the solid tumor XPO1 occupancy assay would most likely include immediate collagenase treatment of biopsies after collection on-site, cryopreservation, and then shipment to a central laboratory. While the assay has not been optimized for solid tumors, it is available for the measurement of XPO1 occupancy in patient PBMCs *ex vivo*. Therefore, the next step of implementing XPO1 occupancy in a clinical trial is to perform the assay with purified cancer cells from hematological patient bone marrow.

Based on our occupancy assay we predict that modulation of pathways downstream of XPO1 may confer resistance to selinexor. Alternatively, resistance to SINE compounds could develop through mutations in the cargo binding pocket of XPO1, i.e. a spontaneous mutation of Cys528 [41]. Since our experiments indicated that the affinity of XPO1 for selinexor is relatively similar across a panel of selinexor sensitive and resistant cell lines, a mutation in the cargo binding pocket of XPO1 is highly unlikely. However, further investigation is warranted to determine how mutations present in the XPO1 cargo binding site might affect compound occupancy. Based on these predicted results, our occupancy assay could be used to identify patients with XPO1 mutations who might not respond to selinexor and could benefit from alternative therapies.

## MATERIALS AND METHODS

### Cell culture and reagents

The following cell lines (ATCC, except where noted) were grown in culture medium supplemented with 10% heat-inactivated fetal bovine serum (FBS, Gibco), 100 units/mL penicillin, 100 μg/mL streptomycin (Gibco), and 1x GlutaMAX (Gibco) (except where noted), and maintained in a humidified incubator at 37°C in 5% CO_2_; Rev-GFP U2OS [McCoy's 5A, 200 ug/ml G418 (Sigma)], MM.1S (RPMI), MV-4–11 (IMDM), THP-1 (RPMI), HCT-116 (McCoy's 5A), AML2 (DSMZ, RPMI), AML3 (DSMZ, RPMI), HT1080 (EMEM), HEL (DSMZ, RPMI), Kasumi-6 (RPMI, 2 mM L-glutamine, 1.5g/L sodium biocarbonate, 4.5 g/L glucose, 10 mM HEPES, 1.0 mM sodium pyruvate, 2 ng/mL GM-CSF, 20% FBS), SINE compound resistant HT1080 (EMEM, 600 nM KPT-185), A549 (RPMI), UCH1 (4:1 IMDM:RPMI), UCH2 (4:1 IMDM:RPMI), LS174T (EMEM), and ASPS-KY (gifted from A. Ogose, RPMI). The XPO1 SINE™ compounds KPT-330 (selinexor), KPT-335, KPT-350, KPT-8602, KPT-301, KPT-9159 (biotinylated LMB, b-LMB), KPT-9058 (biotinylated KPT-276), and KPT-9511 (biotinylated KPT-8602) were synthesized at Karyopharm Therapeutics, Inc. (Newton, MA). LMB was purchased from Cell Signaling.

### Rev-GFP assay

For the typical Rev-GFP assay, U2OS cells stably expressing Rev-GFP [42] were cultured in 96 well plates at 7,500 - 15,000 cells/well. U2OS cells were treated with serial dilutions of selinexor (started at 10 μM; 1:3 dilution), LMB (started at 100 nM; 1:3 dilution), or b-LMB (started at 100 nM; 1:3 dilution) for 4 hours, then collected, washed with PBS, and fixed with 3% paraformaldehyde solution for at least 15 minutes at room temperature. For the wash-out experiment, complete media changed was performed after 4 hours of treatment with selinexor and cells were collected for fixation at 0, 4, 24, 48, 72, and 96 hours post-selinexor wash-out. After fixation cells were stained with DAPI (Invitrogen) for 10 minutes at room temperature. Cells were imaged with a Nikon fluorescent microscope at 10X magnification and intensity of the GFP and nuclear area were measured and recorded. Cells were scored as either GFP nuclear positive or negative. Number of positive cells were divided by total number of cells in order to calculate percentage of cells with nuclear GFP per well. XLFit model 205 was used to calculate IC_50_ curves to report the amount of drug necessary to cause nuclear retention of Rev-GFP in 50% of the cells.

### MTT assay

Cells were seeded in 96-well flat-bottom culture plates. Titrating concentrations of KPT-330, KPT-335, KPT-350, KPT-8602, b-LMB, or leptomycin B (LMB) were added to the wells and incubated at 37°C in a 5% humidified CO_2_ incubator for 72 hours. Triplicate wells per concentration were used to calculate IC_50_ curves. The CellTiter-Fluor Cell Viability Assay (Promega) was performed as instructed by the manufacturer. The whole procedure was repeated three times. The inhibitory rate of cell growth was calculated using the formula: % growth inhibition = (1− OD extract treated)/OD negative control × 100) [43].

### PBMC isolation and treatment

Human donor blood collected in Vacutainer^®^ EDTA tubes (BD) (BioreclamationIVT) was purchased for peripheral blood mononuclear cells (PBMCs) isolation. For dose-response, mice (*n* = 4 per group) were orally administered 0, 0.75, 1.5, 3, or 10 mg/kg selinexor and terminally bled after 4 hours. For time course, mice (*n* = 4 per group) were administered 0, 3, or 10 mg/kg selinexor and terminally bled after 4, 24, 36, 48, 72, and 96 hours. Mouse whole blood was collected in Vacutainer^®^ EDTA tubes (BD) and pooled from each group for each time point. Human and mouse PBMCs were separated by Ficoll (GE Healthcare) gradient. Human PMBCs were cryogenically preserved in Recovery™ Cell Culture Freezing Medium (Gibco). Freshly isolated or previously frozen human donor PBMCs were treated *ex vivo* with titrating concentrations of selinexor for 1 (fresh) or 4 (revived) hours followed by treatment with 1 nM b-LMB for 1 hour and were subsequently collected for processing for the XPO1 occupancy assay. Freshly isolated PBMCs from control or selinexor-treated mice were treated *ex vivo* with 1 nM b-LMB for 1.5 hours prior to collection and processing for the XPO1 occupancy assay.

### XPO1 occupancy assay and Simple Western

Adherent cell lines were plated at 375,000 – 500,000 cells/well in 6 well plates while suspension cell lines were plated at 500,000 – 2,000,000 cells/well and treated with either DMSO (control) or increasing concentrations of KPT-330, KPT-335, KPT-350, KPT-8602, KPT-276, KPT-301, KPT-9058, KPT-9511, b-LMB, or LMB for 1 – 1.5 hours, followed by treatment with either 1 nM b-LMB, 0.1 μM KPT-9058, or 0.1 μM KPT-9511 for 1.5 hours prior to collection and processing. Cells were collected, washed with PBS, and lysed with RIPA Buffer (Thermo Scientific) with protease inhibitors [Complete Ultra Tablets, Mini, EDTA-free, Easy pack (Roche), PhosStop Easy (Roche)]. A portion of the lysate was reserved (input) while the rest was added to washed Dynabeads MyOne Streptavidin T1 (Invitrogen) with RIPA Buffer. Samples were incubated overnight with constant rotation at 4°C. Beads were then washed 5 times with modified RIPA (150 mM NaCl, 50 mM Tris, 1% NP-40, 0.1% Na deoxycholate, 1 mM EDTA, pH 7.8). The reserved portion of lysates (input) and the beads were each mixed with Protein Simple buffer (Protein Simple), DTT (Invitrogen), and fluorescent standards (Protein Simple), then boiled at 95°C for 10 min. Eluates and inputs were analyzed using a capillary western method (Peggy Sue; Protein Simple) with the following primary antibodies: XPO1 (Santa Cruz) and β-actin (Santa Cruz). Capillary westerns were performed by SBH Sciences (Natick, MA). Peak areas corresponding to the protein molecular weight of XPO1 and actin were compared for sample inputs, while the ratio of XPO1 peak areas in eluate to input (ratio of bound or free-fraction XPO1) versus compound concentration were used in XLFit model 205 to calculate 50% and 90% occupancy curves.
